# Abnormal High Body Mass Index among Adolescents of Secondary Schools

**DOI:** 10.31729/jnma.8405

**Published:** 2024-01-31

**Authors:** Mahendra Kumar Agrahari, Mukesh Mallik, Kalyan Sapkota, Ranjita Singh, Suresh Kandel

**Affiliations:** 1 Department of Medicine, Bharatpur Hospital, Bharatpur, Chitwan, Nepal; 2 College of Medical Sciences, Bharatpur, Chitwan, Nepal; 3 Department of Pathology, BP Koirala Memorial Cancer Hospital, Yagyapuri, Bharatpur, Nepal; 4 Purbanchal University School of Health Sciences, Gothgaun, Morang, Nepal

**Keywords:** *children*, *obesity*, *overweight*, *prevalence*, *schools*

## Abstract

**Introduction:**

The rapid increase in the cases of childhood obesity and overweight and its potential to pose other non-communicable diseases has made it a global public health issue. Urbanisation and changing lifestyles also pose a threat to disease in Nepal, but the prevalence of such diseases in Nepal is still not known according to the method recommended by World Health Organization for children's obesity and overweight. This study aimed to find out the prevalence of abnormal high body mass index among adolescents of secondary schools.

**Methods:**

A descriptive cross-sectional study was conducted among children aged 5-19 years at two secondary schools from 7 June to 14 June 2023 after getting ethical approval from the Institutional Review Committee. A convenience sampling method was used. The point estimate was calculated at a 95% Confidence Interval.

**Results:**

Out of 157 children, 29 (18.47%) (12.40-24.54, 95% Confidence Interval) had abnormal high body mass index. Out of 29 children, 19 (65.51%) were male.

**Conclusions:**

The prevalence of abnormal high body mass index in children was found to be higher than other studies done in similar settings.

## INTRODUCTION

Childhood obesity is the state of having excess body fat in children and adolescents, which poses an elevated risk of health complications, impacting a child's well-being. Rising cases of overweight and childhood obesity with an estimation of 340 million globally, have become a pressing challenge to the healthcare world.^1-2^ In Nepal, childhood obesity has surged to 11.2% recently.^3-4^

Although changing lifestyle and social dynamics have been reported as the cause of overweight and childhood obesity,^4^ literature in the Nepalese context for prevalence is limited. Previous studies followed the method of adults rather than children for BMI calculation. This created the need for improved studies showing adherence to BMI-for-age criteria as per WHO recommendations for children.^5^

This study aimed to find out the prevalence of abnormal body mass index among children aged 5-19 years of secondary schools.

## METHODS

A descriptive cross-sectional study was conducted among the private school of Gaindakot Municipality and Bharatpur Metropolitan City, Chitwan, Nepal from 7 June to 14 June 2023 after obtaining ethical approval from the Institutional Review Committee, Bharatpur Hospital, Chitwan, Nepal (Reference number: 079/80019). Schoolchildren from grades 7 to 10 whose parents expressed verbal consent for their children's participation, and students who agreed to provide information were included in the study. Any differently abled children, children with a previous history of metabolic disorders, or mental disabilities were excluded from the study. A convenience sampling method was used. The sample size was calculated using the following formula:


$$\begin{array}{ll} \text{n} & ={\text{Z}}^{2}\times \frac{\text{p}\times \text{q}}{{\text{e}}^{\text{2}}}\\ & =1.96^{2}\times \frac{0.50\times 0.50}{0.08^{2}}\\ & =150 \end{array}$$

Where,

n = minimum required sample size
Z = 1.96 at 95% Confidence Interval (CI)
p = prevalence taken as 50% for maximum sample size
q = 1-p
e = margin of error, 8%

The calculated sample size was 150. However, 157 children were included in the study.

BMI for age was extracted by importing the necessary data in the WHO AnthroPlus Software as recommended by WHO for children aged 5 to 19 years. Thereafter, as per the criteria of WHO, BMI for age was categorized into three categories i.e. overweight, obese and severely obese.^5^

Data was entered and analyzed using Microsoft Excel 2007. The point estimate was calculated at a 95% CI.

## RESULTS

Out of 157 children, 29 (18.47%) (12.40-24.54, 95% Confidence Interval) had abnormal high BMI. Among them, 23 (79.31%) were overweight (Table 1).

**Table 1:** Frequency of abnormal high BMI (n=29).

Category	n (%)
Overweight	23 (79.31)
Obese	4 (13.79)
Severely obese	2 (6.90)

**Figure 1:**
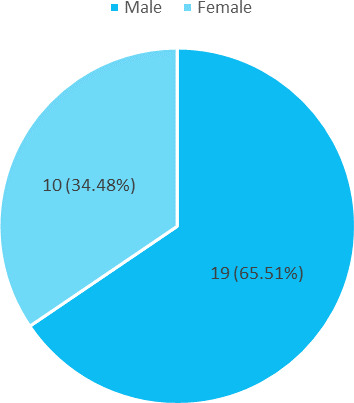
Gender distribution (n= 29).

Out of 29 children, 19 (65.51%) were male (Figure 1)

Most of the children, 26 (89.66%) were Brahmin/Chettri. Twenty-two (75.86%) belonged to a joint family. Sixteen (55.17%) children had business as the occupation of their father. Similarly, 16 (55.17%) children had their occupation of the mother as a homemaker (Table 2).

**Table 2:** Frequency tables of data description of Socio-demographic variables (n= 29).

Variable	n (%)
**Age**	
12	6 (20.7)
13	9(31.0)
14	6 (20.7)
15	6 (20.7)
16	2 (6.9)
**Ethnicity**	
Brahmin/Chhetri	26 (89.66)
Janajati	2 (6.90)
Madheshi	1 (3.45)
**Family type**	
Joint	7 (24.14)
Nuclear	22 (75.86)
**Religion**	
Hindu	29 (100)
**Number of siblings**	
Zero	5 (17.24)
One	17 (58.62)
Two	4 (13.79)
Three or more	3 (10.34)
**Occupation of father**	
Foreign employment	2 (6.90)
Agriculture	3 (10.34)
Job	8 (27.59)
Business	16 (55.17)
**Occupation of mother**	
Foreign employment	2 (6.90)
Agriculture	2 (6.90)
Business	8 (27.59)
Homemaker	16 (55.17)
Job	1 (3.45)

## DISCUSSION

Out of 157 children, 29 (18.47%) had abnormal high BMI. The prevalence of high abnormal BMI was higher than the studies done in similar settings. The overall prevalence of overweight in children was found to be 6.1% in a study conducted among school children of Kathmandu and Bhaktapur which is lower than the findings of our study.^6^ The result of another study conducted in a similar setting found the prevalence of overweight to be 9.8% which is lower than our findings.^7^ The prevalence of overweight and obesity among adolescents in the higher secondary schools of the Kaski district was 8.1% in a previous similar study.^8^

We followed BMI-for-age which is the recommended indicator for the study population of children of age 10-19 years.^9^ This study was carried out to find the prevalence of childhood obesity and overweight because of changing lifestyles, affection towards junk foods and attraction towards emerging technologies making life sedentary. The topic is important because childhood obesity poses an elevated risk for future health complications such as diabetes, heart diseases, physical and psychosocial problems and other non- communicable diseases in adulthood.^1-2,10^

In our study, the prevalence of high abnormal BMI was found more in males than in females which is opposite to findings of other previous studies of similar settings.^8,11-14^

Other than finding the prevalence across sex, we also found proportions of high abnormal BMI of children across their family type, and number of siblings as these factors are reported to affect the outcome.^7^

## CONCLUSIONS

The prevalence of high abnormal BMI in children was found to be higher than other studies done in similar settings. The results of this study can be taken as evidence for further study in the future.
